# Efficacy predictors of a second tumor necrosis factor inhibitor in the treatment of rheumatoid arthritis

**DOI:** 10.1097/MD.0000000000021827

**Published:** 2020-08-28

**Authors:** Jennifer Reams, Andrea Berger, Alfred Denio

**Affiliations:** Geisinger Medical Center, Danville, PA, USA.

**Keywords:** rheumatoid arthritis, tumor necrosis factor inhibitor

## Abstract

To retrospectively evaluate initial tumor necrosis factor inhibitor (TNFi) failure patients for clinical predictors of response to a 2nd TNFi in our 4282 rheumatoid arthritis (RA) patient database.

A cross-sectional retrospective manual chart review of the electronic health record (EHR) was performed on 322 “real world” RA patients who were prescribed 2 TNFis. Response to TNFi was determined by the treating provider who had real time Clinical Disease Activity Index (CDAI) scores to inform treatment decisions. Age, gender, body mass index (BMI), insurance provider, duration of disease, cyclic citrullinated peptide antibody (CCP) and rheumatoid factor (RF) positivity, concomitant disease modifying anti-rheumatic drug therapy, length of time between diagnosis and start of 1st and 2nd TNFi, transient efficacy of 1st TNFi (defined as response to TNFi at 3 months but later lost response), and reason for discontinuation of 1st TNFi were analyzed. A multivariable logistic regression model was used to model response to a 2nd TNFi.

Response proportions to the 2nd TNFi were greater in females (161/223, 72.2% response female vs 41/75, 54.7% male, *P* < .01), those who began their 1st TNFi within 3 months of their RA diagnosis, and in RF+ patients (123/170, 72.4% response seropositive vs 66/110, 60.0% seronegative, *P* < .03). The higher female response rate was independent of age, BMI, and seropositivity.

In RA patients who failed an initial TNFi, female patients and patients with RF+ were more likely to have a clinical response to a 2nd TNFi. In the absence of these predictors, stronger consideration for choosing a biologic with an alternative mechanism of action might be given when the 1st TNFi fails.

## 1 Introduction

Dramatic improvement in the treatment of rheumatoid arthritis (RA) with targeted biologic agents has made disease remission a realistic goal. Included in these agents are TNFis which have improved disease outcomes. Not every patient responds similarly to the same medications, however. The response rate of TNFis in RA is variable and to some extent unpredictable, making treatment decision-making quite complex. What further complicates the treatment picture is the continual development and evolution of additional targeted therapies to include not only TNFi's, but also T-cell co-stimulation modulators, Janus kinase (JAK) inhibitors, interleukin-6 (IL-6) inhibitors, and B-cell depletors. While the 2015 American College of Rheumatology guidelines for the treatment of RA suggest that all biologic disease-modifying antirheumatic drugs (DMARDs) are reasonable considerations after the failure of a traditional DMARD, some insurance carriers require RA patients to have failed at least two different TNFi agents prior to a trial of a biologic agent with an alternate mechanism of action (MOA).^[1]^

It has been suggested through previous study that adequate clinical response to a 2nd TNFi after failing a 1st TNFi occurs 40% to 50% of the time.^[2,3]^ Further evidence, however, suggests that response rates to a non-TNFi biologic agent after first failing a TNFi may be superior to a trial of a 2nd TNFi with 60% to 70% efficacy in achieving a good or moderate response as compared to 40% to 50% efficacy when using a 2nd TNFi.^[4]^ Health care costs are also influenced by physician treatment decisions. Medical and pharmacy claims data have shown that the health care cost per patient is lower for those who have been switched from a TNFi to an agent with an alternative MOA as compared to those who are cycled within TNF inhibitors.^[5]^

We aimed to assess the current RA treatment algorithms mandated by our local prescription drug plans (PDPs) which were dominated by PDP step edits that required failure of two TNFis before a biologic with an alternative MOA would be authorized. In those who have failed to respond adequately to an initial TNFi, we looked for clinical predictors of response to a 2nd TNFi.

## 2 Methods

The ethical approval was waived or not necessary as the study protocol was reviewed by the Geisinger Institutional Review Board and was not determined to be subject to research regulations under the Federal Common Rule. It did not meet the specific definition of research under 45 CFR 46.102(d) put forth by the US Department of Health and Human Services.

We retrospectively analyzed our 4282 validated RA patient database electronic health record (EHR) and performed manual EHR chart reviews on 322 RA patients seen over the course of 4 years who were noted to have been prescribed more than one TNFi. Our RA population was over 90% Caucasian with a primarily low to middle income economic demographic. The general population is thought to be relatively stable compared to other demographic areas, although this has not been formally studied. There were 13 staff rheumatologists, 4 rheumatology fellows and 2 nurse practitioners spread across rural central Pennsylvania seeing RA patients in our system during this period. At each clinic visit, patients were asked to complete an electronic data questionnaire which was designed to collect Clinical Disease Activity Index (CDAI)-relevant information.

In our chart abstraction, age, gender, body mass index (BMI), insurance provider, duration of disease, cyclic citrullinated peptide antibody (anti-CCP) and rheumatoid factor (RF) positivity, concomitant disease modifying anti-rheumatic drug (DMARD) therapy, length of time between diagnosis of RA and start of 1st and 2nd TNFi, reason for drug discontinuation, and efficacy of 1st and 2nd TNFi as evidenced by sustained or transient reduction in either CDAI or treatment response judgement of the treating provider when CDAI data was not available were all recorded. Transient TNFi response was defined as initial response as assessed by the treating provider at 3 months with later loss of response. Formal CDAI cutoffs to define “response” were not established as the impression of the treating provider took precedence in this “real world” study. The provider was not required to adhere to formal guidelines regarding CDAI-determined achievement of low disease activity or remission.

Patients who did and did not respond to their 1st and 2nd TNFi were compared using Pearson's chi-square or Fisher's exact tests and Student's *t* tests or Wilcoxon rank-sum tests. A multivariable logistic regression model that included gender, age, BMI, and seropositivity was used to model response to a 2nd TNFi.

## 3 Results

Baseline demographic characteristics are listed in Table 1. Data was derived from a database native to a health system located in rural central Pennsylvania, consisting of predominately Caucasian patients. The mean age of patients at baseline was 56 years, while 74.8% were women and 25.2% were men. The mean BMI of all patients was 31.4, placing them in an obese demographic. Those who were privately versus federally insured were 54.7% and 45.3%, respectively (not included in table). 43.8% of patients were positive for cyclic citrullinated peptide (anti-CCP) and 43.8% of patients were negative for anti-CCP with 12.4% unknown. A majority of patients had a positive RF at 58.1% while 35.7% were RF negative with 6.2% unknown. Additionally, 82.3% of patients were prescribed methotrexate at the time of the study and 62.7% were prescribed an alternative traditional DMARD. The median CDAI at the time of the first TNFi initiation was 23. CDAI was the predominate means to assess disease activity among the treating providers of this cohort of patients. It was routinely and systematically collected at the time of routine patient visits via electronic questionnaires. CDAI was successfully collected and documented in 50% or more of office visits in 79% of our RA patient population.

**Table 1:**
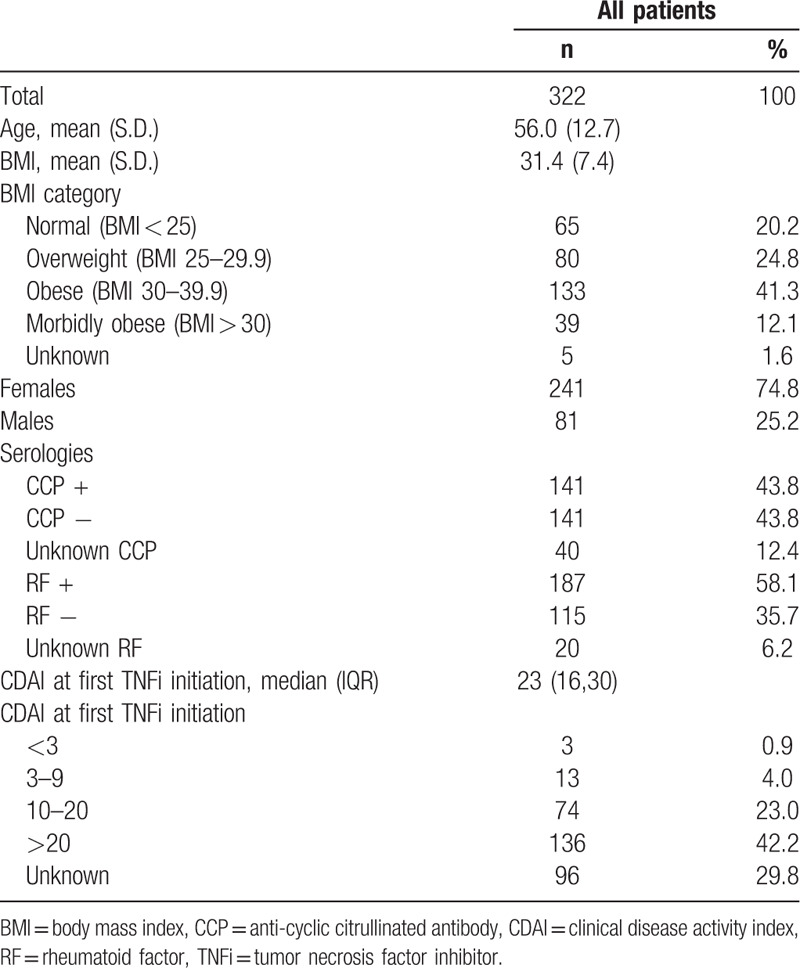
Baseline characteristics.

Whether there was no response or transient achievement of response to a 1st TNFi as measured by CDAI or opinion of the treating provider is displayed in Table 2. All responses to the 1st TNFi were transient by definition; all patients were eventually prescribed a 2nd TNFi due to lack of efficacy or intolerance to the first TNFi. RF positive patients were more likely to have transiently responded to the 1st TNFi than RF negative patients (144/183, 78.7% transient response when RF positive vs 72/114, 63.2% transient response when RF negative, *P* < .01). Whether there was a transient response or not could not be determined in 5 patients (1.6%) due to lack of clear medical record documentation. Age, BMI, insurance provider, gender, anti-CCP status, concomitant DMARD therapy (not included in table), and disease duration prior to initiation of the first TNFi did not reach statistical significance when analyzed for impact on transient efficacy versus no efficacy.

**Table 2:**
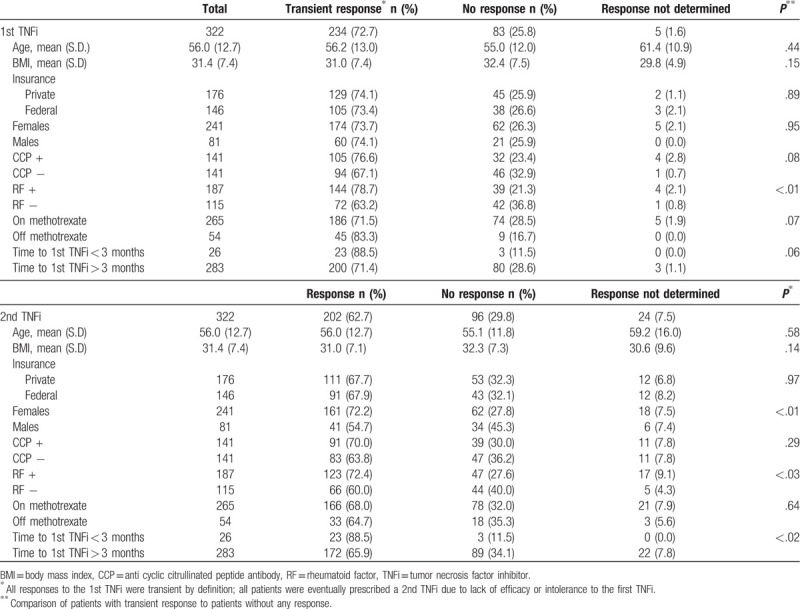
Response to 1st and 2nd tumor necrosis inhibitor (TNFi) based on demographic and clinical data.

Responses to the 2nd TNFi are displayed in Table 2. Response to the 2nd TNFi could not be determined in 24 patients (7.5%) due to incomplete documentation in the medical record. Response rates to the 2nd TNFi were greater in females versus males (161/223, 72.2% response if female vs 41/75, 54.7% response if male, *P* < .01) and in RF positive vs RF negative patients (123/170, 72.4% response if RF positive vs 66/110, 60.0% if RF negative, *P* = .03). The predilection for female response was independent of age, BMI, and seropositivity. In a multivariable model including age, sex, BMI, and seropositivity, females were still more likely than men to show a response to the 2nd TNFi (OR 2.26; 95% CI for OR 1.26, 4.05, *P* < .01), and RF positive patients were still more likely to show a response than negative patients (OR 1.81; 95% CI for OR 1.07, 3.07, *P* = .03). If the time to first TNFi was 3 months or less from initial diagnosis of RA, sustained response to 2nd TNFi was more likely compared to longer times (23/26, 88.5% if 1st TNFi started <3 months within initial RA diagnosis vs 172/261, 65.9% if 1st TNFi started >3 months within initial RA diagnosis, *P* < .02).

Transient response of the 1st TNFi based on agent used and BMI category is delineated in Table 3. All responses to the 1st TNFi were transient by definition; all patients were eventually prescribed a 2nd TNFi due to lack of efficacy or intolerance to the 1st TNFi. Etanercept and adalimumab were the most common TNF inhibitors prescribed due to local carrier formulary step edits. Infliximab was infrequently used, with only 13 users total across all weight categories. Weight-independent dosed agents did not have greater transient efficacy than weight-dependent dosed agents.

**Table 3:**
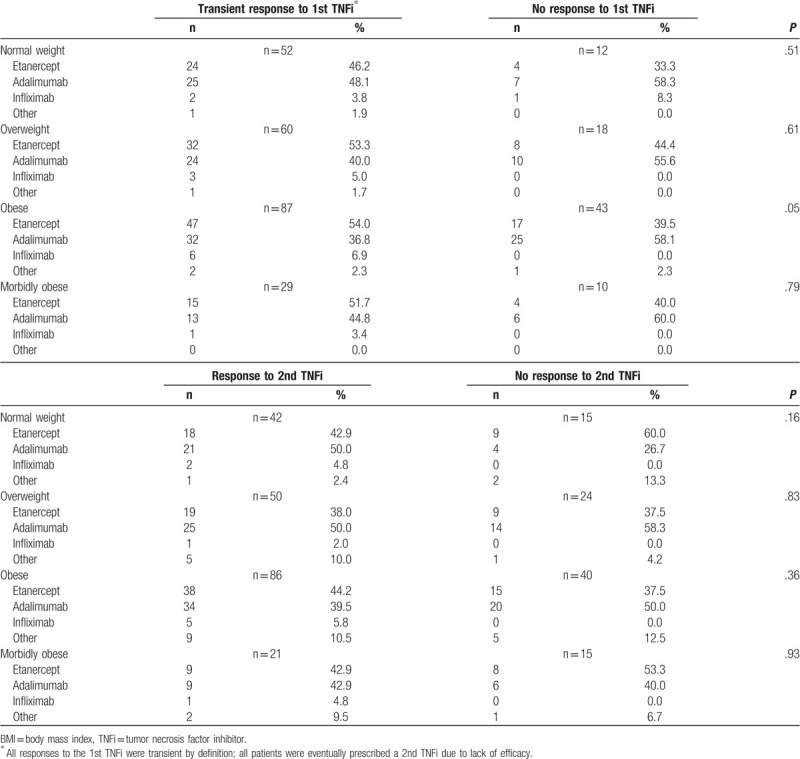
Response to 1st and 2nd tumor necrosis inhibitor (TNFi) based on agent used and BMI category.

Response of the 2nd TNFi based upon agent used and BMI category is delineated in Table 3. Etanercept and adalimumab remained the most common TNF inhibitors used. Infliximab was used in a total of 9 patients. Weight-independent agents did not have a statistically greater transient efficacy than weight-dependent agents, although it should be noted that all 9 patients treated with infliximab as a 2nd TNFi responded to the therapy. Statistical analysis was limited by the low number of patients on infliximab.

Reason for discontinuation of 1st TNFi (lack of efficacy vs intolerance) did not affect the 2nd TNFi response rate (data not shown).

## 4 Discussion

The present observational cross-sectional cohort “real world” study of 2nd TNFi response rates found superior response in women, RF positive patients, and patients starting their 1st TNFi therapy in the first 3 months of disease. Gender has frequently been studied in regards to its role in predicting remission, disease progression, and treatment response in RA.^[6–11]^ Being female in itself appears to be an independent risk factor for lack of attainment of remission as defined by Disease Activity Score 28 (DAS28) scores despite similar initial disease progression at time of diagnosis and subsequent treatment as their male counterparts.^[6]^ Differences in gender response rates may be influenced by which disease activity measure is used.^[7]^ Maynard et al in a Veterans Administration patient population of primarily male RA patients found that the DAS28-ESR measure was more favorable to men achieving remission or low disease activity, but was not seen in CDAI, Rapid 3, or DAS28-CRP. This suggests that sex differences in ESR alone may be causing the DAS28-ESR to be falsely elevated in women. Jawahreer et al observed that despite similar treatments, women consistently have worse disease progression as defined by DAS28-ESR, physician global scores, and tender joint counts at 2-year follow-ups.^[8]^ Despite similar objective radiographic joint destruction, women as compared to men show high pain perception as measured with DAS28 and Health Assessment Questionairre (HAQ).^[9]^ These disease parameters have been further studied as they relate specifically to TNFi therapy. Not only do men achieve a more favorable treatment response than women in early RA, but they achieve this response earlier in their treatment course.^[10]^ This gender disparity in time to treatment response was not found in those with established RA, however.^[10]^ All of these prior findings may help explain the patterns of our own findings in the present study.

Again, our study showed that a second TNFi was more beneficial for those who were female. One hypothesis as to why this may be the case is that more men may have responded to the first TNFi and did not require a 2nd TNFi trial. Instead, a more refractory RA phenotype would therefore have received a 2nd TNFi. Another possible explanation might be the evidence that women may take longer to respond to treatment.^[10]^ A longer total TNFi treatment course for women is achieved with switch to a 2nd TNFi.

Seropositivity as a marker for a more favorable response rate to TNFis seems to contradict much of the literature in this area. Santos-Moreno at al, for example, found that RF positivity predicts lower TNFi response rates than RF negativity in their small observational cohort.^[11]^ A recent meta-analysis of 14 studies by Lv et al revealed, however, that in “non real-world” studies there is no predictive value of RF in response to any TNFi in RA patients.^[12]^ It is possible that more of the RF negative patients responded to the 1st TNFi while leaving more treatment-refractory RF negative patients to be given a trial of a 2nd TNFi. Although our RA patient database has been validated, the low CCP antibody incidence would raise possibility that some of the seronegative patients may not have RA which might explain lower 2nd TNFi response rates in this group.

The strength in this study lies in its size, covering a 4282 validated RA patient cohort with comprehensive chart review of the 322 patients who met inclusion criteria of having been prescribed two or more TNF inhibitors. To the best of our knowledge, a study of this size has not been performed to address potential clinical factors influencing why some patients fail a trial of a 2nd TNFi while others do not. While the “Switch” study attempted to address whether or not switching to an alternate TNFi after failing an initial TNFi is advantageous as either a switch to abatacept or rituximab, the result was not clear. Reduction of global assessment of pain was seen in all involved treatment groups irrespective of second-line agent used.^[13–15]^ Our study was a real-world outcome study where all the psychosocial and economic factors can and do affect outcome but cannot be easily controlled or mitigated. We think the stable nature of our RA population accounts for the low numbers where TNFi response could not be determined.

Despite the power and size of this study, several limitations likewise exist. Manual chart collection and review can lead to subjective interpretation of provider progress notes where there may be much less clarity than desired for data extraction. Similarly, reporting of CDAI was not universal among all treating providers in this study. The result is, in some instances, incomplete objective data delineating whether or not each TNFi resulted in a reduction in disease activity. We also did not examine ESR or CRP data or systematically determine low disease activity or remission rates by CDAI to define adequate response. The vast majority of the time, however, the treating providers had real time CDAIs to obtain a sense of disease activity and inform treatment decisions. We also did not individually adjust for age, race, smoking status, or disease duration. While the differences in 2nd TNFi response rates between men and women and between seropositive and seronegative patients are not great, they are clinically significant given the cost and duration of time lost when patients fail to respond to a 2nd TNFi. Nevertheless, it cannot be concluded from this study that seronegative male patients switching to a biologic with alternative mechanism of action rather than a 2nd TNFi would have had better response rates. It was surprising that the reason for failure of the 1st TNFi (drug intolerance vs lack of efficacy) did not affect 2nd TNFi treatment response.

There may be other predictors of 2nd TNFi response that we did not examine. For instance, there is evidence that RA patients with higher comorbidity index scores will not respond as well to biologic therapies although the differences were small for TNFi's.^[16]^ We did not extract comorbid health problems in our manual chart reviews. It would have been quite challenging to collate all the comorbid diseases with graded severity in a large retrospective electronic chart review. Although our RA patient database has been validated in regards to diagnosis of RA, the associated comorbid health problems have not been validated.

It must also be noted that our local drug prescription plans mandated the use of either adalimumab or etanercept as first and second line biologics in the treatment of RA. This resulted in comparatively fewer patients on alternate TNF inhibitors, including infliximab. The sequencing of prescriptions between non weight-based TNFi did not statistically influence efficacy. While our data suggests a possible trend toward greater efficacy of the weight-based infliximab as a 2nd TNFi in those with higher BMIs, our data set is under-powered to draw any definite conclusions. Of note, all 9 patients who received infliximab as a 2nd TNFi responded favorably to the medication. This finding leaves room to consider that patient weight is a critical clinical predictor of 2nd TNFi efficacy. Future study should be aimed at better-addressing this important clinical question.

In conclusion, in RA patients who failed to achieve or sustain a clinical response to an initial TNFi, female patients, patients with positive RF, and patients whose diagnosis of RA was within three months of the 1st TNFi initiation were more likely to have a clinical response to a 2nd TNFi agent. In the absence of these criterion, our data suggest a significantly lower response rate to a 2nd TNFi. In these individuals, stronger consideration might instead be given to a biologic with an alternative MOA and PDPs may want to consider more nuanced considerations in their prior authorization processes.

## Acknowledgments

Philip Dunn, DO, Eva Rottmann, DO, William Torelli, DO, Jason Bankert, DO, Muhammad Bashir, MD for assistance in the manual chart review data extraction.

The data that support the findings of this study are available from the corresponding author, [AD], upon reasonable request.

## Author contributions

**Conception and design of the study:** Alfred Denio, Jennifer Reams.

**Data collection:** Jennifer Reams.

**Data analysis and interpretation:** Andrea Berger, Alfred Denio, Jennifer Reams.

**Drafting the article:** Jennifer Reams.

**Critical revision of the article:** Alfred Denio.

**Final approval of the version to be published:** Jennifer Reams, Andrea Berger, Alfred Denio.
